# Influence of strengthened hemoperfusion combined with continuous venovenous hemofiltration on prognosis of patients with acute paraquat poisoning: SHP + CVVH improve prognosis of acute PQ patients

**DOI:** 10.1186/s40360-020-00428-z

**Published:** 2020-07-06

**Authors:** An-bao Chen, Fang Li, E-mu Di, Xiao Zhang, Qun-yuan Zhao, Jing Wen

**Affiliations:** Department of Emergency Medicine, the Second Affiliated Hospital of Kunming Medical University, Kunming, China

**Keywords:** Strengthened hemoperfusion, Continuous venovenoushemofiltrations, Acute paraquat poisoning, Acute respiratory failure

## Abstract

**Background:**

The success rate of rescue is extremely low in acute paraquat poisoning. This study aimed to assess whether strengthened hemoperfusion (SHP) combined with continuous venovenous hemofiltration (CVVH) improves prognosis in patients with acute paraquat poisoning.

**Methods:**

Patients from January 2005 to December 2018 were enrolled retrospectively. All selected patients were administered conventional therapy. They were divided according to the received treatments in the conventional therapy, hemoperfusion (HP), CVVH, SHP and SHP + CVVH groups. Follow-up was implemented until the 90th day after poisoning. Other outcomes included all-cause mortality on the 15th day after poisoning, and the percentages of respiratory failure and mechanical ventilation use.

**Results:**

The study included 487 patients,and 211 died in all. Mortality rate in the SHP + CVVH group on the 90th day after poisoning was significantly decreased compared with those of other groups (*p*<0.001). Survival curves of all groups showed significant differences (*p*<0.001). SHP combined with CVVH was an independent factor reducing mortality risk (*p*<0.001). Mortality rate in the SHP + CVVH group on the 15th day after poisoning was also significantly decreased (*p* < 0.05). The proportions of patients in the SHP + CVVH group with acute respiratory failure and mechanical ventilation were significantly lower than those of other groups (*p* < 0.05).

**Conclusions:**

SHP with CVVH may decrease the mortality rate of patients with acute paraquat poisoning on the 90th day after poisoning and improve the prognosis.

## Background

Paraquat is an herbicide widely applied in many countries due to low price and good weeding effects. However, the poisonous dose of acute paraquat is low and the fatality rate reached up to 50–90% [1].It has a broad hazard and influence scope. There is currently no antidote with specific effects on acute paraquat poisoning. Comprehensive therapies such as reducing poison absorption, eliminating the absorbed poison, anti-inflammatory factor, symptomatic and supportive treatments and others are mostly adopted clinically.

Multiple clinical trials have shown that hemoperfusion (HP) can effectively eliminate paraquat absorbed into the blood [2]. Early and single or discontinuous and repeated hemoperfusion sessions are mostly adopted to treat acute paraquat poisoning in current clinical studies [3–5]. Continuous venovenous hemofiltration (CVVH) effectively suppresses proinflammatory cytokines, including interleukin (IL)-1β, IL-6 and tumor necrosis factor alpha (TNFα), and has been used to treat multiple organ dysfunction and septic shock [6, 7]. Inflammatory reactions may be another important mechanism by which acute paraquat poisoning causes organ damage [8]. Theoretically, CVVH can be used to treat and cure patients with acute paraquat poisoning. According to few single-center clinical research studies, the influence of early and single or discontinuous and repeated HP can be combined with CVVH on prognosis of patients with acute paraquat poisoning [4, 9]. In acute paraquat poisoning, HP combined with CVVH might assist in maintaining electrolytes and acid-base balance, and in better removal of inflammation factors, creatinine and urea nitrogen [3].

Paraquat may be stored in muscles and lungs for weeks to months. Paraquat could be released into the blood again if the blood concentration drops, causing further damage [3]. Animal experiments revealed that early strengthened hemoperfusion (SHP) can significantly improve the survival rate 30 days after poisoning of animals with acute paraquat poisoning, while single HP showed no improvement in the survival at 30 days after paraquat poisoning [10]. Clinical study on the effect of SHP combined with CVVH on the prognosis of acute paraquat poisoning has not been reported yet.

To address these knowledge deficits, we aimed to investigate whether SHP combined with CVVH improved the prognosis of patients with acute paraquat poisoning.

## Methods

### Ethics

The research was approved by the Ethics Committee of the Second Affiliated Hospital of Kunming Medical University and conformed to related requirements of Declaration of Helsinki. Informed consent was exempted by the ethics committee of the second affiliated hospital of kunming medical university.

### Conditions of selected patients

Patients admitted to the emergency department of the second affiliated hospital of kunming medical university from January 2005 to December 2018 were enrolled retrospectively.

Inclusion criteria were as follows: 1) patients no less than 14 years old; 2) clear history of paraquat contact; 3) treatment in the Emergency Department of the Second Affiliated Hospital of Kunming Medical University within 48 h after poisoning; 4) patients should have an expected survival time of > 24 h after admission.

Exclusion criteria were as follows: 1) mixed poisoning; 2) pregnancy; 3) chronic lung disease, chronic liver and kidney function impairment; 4) missing a follow-up visit.

### Grouping

Patients are divided into 5 groups according to different therapies: conventional therapy (control), hemoperfusion (HP), continuous venovenous hemofiltration (CVVH), strengthened hemoperfusion (SHP), strengthened hemoperfusion combined with continuous venovenous hemofiltration (SHP + CVVH) groups.

### Therapies

For the conventional therapy method, gastric lavage with 2% sodium bicarbonate immediately after hospital admission, provide 20% mannitol for catharsis followed by montmorillonite powder for adsorption treatment; in addition, antioxidants, immunosuppressors, antagonists, anti-inflammatory drugs, and symptomatic and supportive treatments were administered. All patients underwent conventional therapy. Based on this, the hemoperfusion method in the hemoperfusion group was performed as follows: the femoral vein was adopted as puncture position. Then,low molecule heparin anticoagulation or non-heparin methods were adopted for anticoagulation methods according to the conditions of patients condition. Hemoperfusion treatment was administered within 1 h after hospital admission on a JF-800A instrument (Jafron, Zhuhai, China). Perfusion (2 h/session) for 1–3 times was implemented according to the wishes of patients and families. In the process of hemoperfusion, blood guiding speed was 150–180 ml/min.In the strengthened hemoperfusion method, hemoperfusion was performed for 5 times continuously within 1 h after hospital admission on a JF-800A instrument (Jafron, Zhuhai, China). Each perfusion lasted 2 h, for a total hemoperfusion time of 10 h. Puncture position, blood guiding speed and anticoagulation methods were as described for the hemoperfusion group. For continuous venovenous hemofiltration, continuous venovenous hemofiltration was performed after consent by families upon hospital admission on a Prismaflex, CRRT machine (Jinbao, Germany). The displacement fluid was basic displacement liquid of hemofiltration (4000 ml; Sichuan, China). Low molecular heparin anticoagulation, citric acid anticoagulation or non-heparin methods were adopted for anticoagulation according to patient condition. Continuous hemoperfusion and continuous hemofiltration adopted in the continuous hemoperfusion + continuous hemofiltration group were same as described above. Hemofiltration was administered for 5 continuous times within 1 h after hospital admission, and continuous hemofiltration therapy followed perfusion.

### Estimation method of poisonous dose

A sip of poison taken corresponded to 5 ml approximately, while a swig was recorded as 20 ml approximately [11].

### Outcomes

The main outcome was all-cause mortality, i.e. the death rate from all causes, on the 90th day after poisoning. Other outcomes included mortality rate 15 days after poisoning, and the percentages of patients with respiratory failure and mechanical ventilation use after poisoning.

### Data collection and follow-up

The method of telephone follow-up was adopted to trace the prognosis of patients in the research.

### Statistical analysis

SPSS23.0 for windows (IBM, Armonk, NY) was used for statistical analysis. Continuous data were tested for normality using the Kolmogorov–Smirnov test. Continuous variables of baseline characteristics in all groups were represented by mean ± standard deviation (mean ± SD). One-way analysis of variance was performed for group comparison. The Kruskal-Wallis H test was performed for multi-group comparisons of quantitative data, with post-hoc Dunn’s test for multiple group pair comparisons. Classification variables were represented as percentage, and the Chi-square test was carried out to compare qualitative data in multiple groups, with Bonferroni correction for subsequent multiple group pair comparisons. *P*<0.005 was considered as statistically significant difference. The Kaplan-Meier method was adopted to compare survival among groups, and the Log-Rank was used to assess differences. Univariate and multivariate COX regression analyses (calculating hazard ratios [HRs]) were performed for assessing 90-day survival; parameters with *P* < 0.05 were included in multivariate analysis. *P* < 0.05 indicated statistical significance.

## Results

### Characteristics of the included patients

**Fig. 1:**
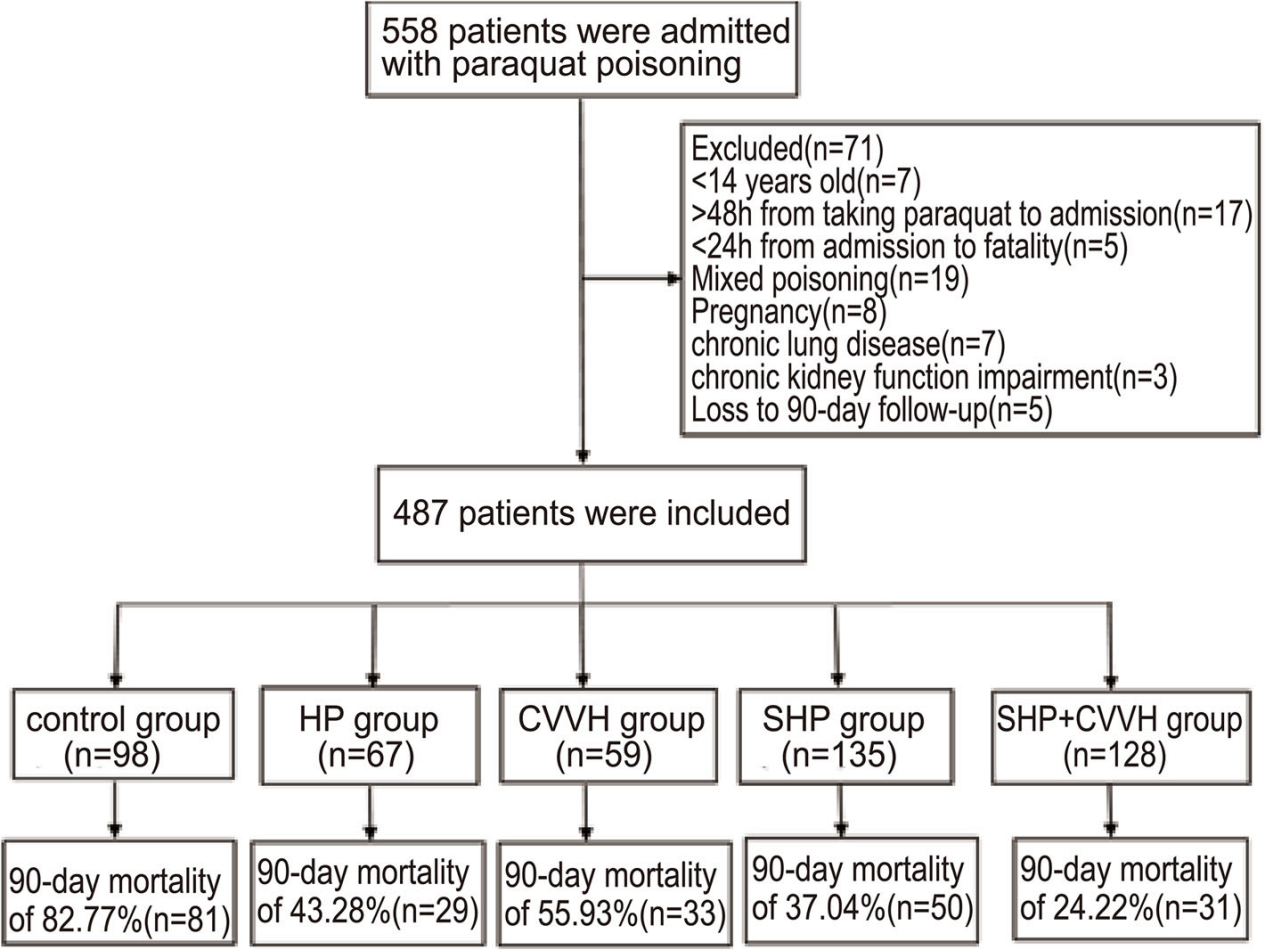
Flowchart of patients. HP, hemoperfusion. CVVH, continuous venovenous hemofiltration. SHP, strengthened hemoperfusion

A total of 558 patients with acute paraquat poisoning treated in the Emergency Department of the Second Affiliated Hospital of Kunming Medical University from January 2005 to December 2018 are included in the research. Among these, 29 patients did not meet the entry criteria, and 42 met the exclusion criteria. Therefore, a total of 487 patients were enrolled. Ninety-eight patients (20.12%) were in the control group, 67 patients (13.76%) in the HP group, 59 (12.11%) in CVVH group, 135 (27.72%) in SHP group, and 128 (26.28%) in SHP combined with CVVH group were enrolled (Fig. 1). There were no sigificant differences in baseline characteristics among all groups (see Table 1 for details). There were 433 (88.91%) patients with oral poisoning, including 89 (18.28%) patients from the control group, 61 (12.53%) from HP group, 113(23.20%) patients from SHP group, 53 (10.88%) patients of CVVH group and 117(24.02%) from SHP + CVVH group. The dose of oral poisoning of all groups were 43.70 ± 19.03 ml, 45.04 ± 18.04 ml, 43.07 ± 21.04 ml, 45.17 ± 17.83 ml and 44.91 ± 17.92 ml, respectively. There were no significant differences among the groups (*p* = 0.62). Time from poisoning to HP in HP group, SHP group and SHP + CVVH group was 9.12 ± 2.36 h, 8.96 ± 2.59 h and 9.02 ± 2.75 h. The difference among the groups had no statistical significance (*p* = 0.89).

**Table 1:** Basic characteristics of selected patients

Indexes	control (*n* = 98)	HP (*n* = 67)	CVVH (*n* = 59)	SHP (*n* = 135)	SHP + CVVH (*n* = 128)	*P*
Age (years)	35.88 ± 15.77	38.93 ± 14.34	32.41 ± 16.02	33.27 ± 13.03	35.97 ± 14.08	0.61
Male, n (%)	43(43.88%)	27(40.30%)	25(42.37%)	56(41.48%)	54(42.19%)	0.993
Body mass index	23.07 ± 4.10	22.31 ± 5.22	24.13 ± 3.30	23.94 ± 4.07	22.01 ± 5.44	0.39
Number of people with oral poisoning, n (%)	89(90.82)	61(91.04)	53(89.83)	113(91.11)	117(91.41)	0.263
Time from poisoning to treatment (h)	7.03 ± 3.47	6.54 ± 4.05	7.19 ± 3.71	6.35 ± 3.09	6.73 ± 3.41	0.17
Time from poisoning to gastric lavage (h)	4.49 ± 1.31	4.08 ± 1.72	4.45 ± 1.14	4.37 ± 1.38	4.19 ± 1.49	0.871
Time from poisoning to hemoperfusion (h)		9.12 ± 2.36		8.96 ± 2.59	9.02 ± 2.75	0.89
Acute kidney injury, n (%)	26(26.53%)	15(22.39%)	14(23.73%)	32(23.37%)	35(27.34%)	0.923
BUN(mmol/L)	7.33 ± 5.08	7.21 ± 5.14	7.47 ± 4.89	7.41 ± 4.73	7.50 ± 4.55	0.94
CREA (μmol/L)	129.00 ± 98.52	127.91 ± 91.55	133.04 ± 87.05	136.34 ± 90.34	134.51 ± 92.74	0.77
AST (IU/L)	79.04 ± 52.8	78.13 ± 61.77	79.26 ± 50.41	77.45 ± 54.73	78.24 ± 57.49	0.59
ALT (IU/L)	90.74 ± 57.82	88.30 ± 61.37	91.04 ± 53.33	88.21 ± 60.32	89.36 ± 55.82	0.74
Respiratory failure, n(%)	13(13.27%)	7(10.45%)	6(10.17%)	19(14.07)	21(16.41%)	0.726
Arterial blood gas analysis						
PH	7.38 ± 0.14	7.31 ± 0.17	7.40 ± 0.11	7.35 ± 0.18	7.42 ± 0.17	0.83
PO_2_ (mmHg)	84.05 ± 19.44	83.42 ± 18.79	83.14 ± 16.94	85.74 ± 17.03	83.97 ± 17.28	0.74
PCO_2_ (mmHg)	26.63 ± 11.05	26.08 ± 12.07	29.15 ± 10.85	27.46 ± 11.33	27.04 ± 13.01	0.80

All clinical data were collected at the time of admission

### Reduction of mortality rate in patients by SHP combined with CVVH

**Fig. 2:**
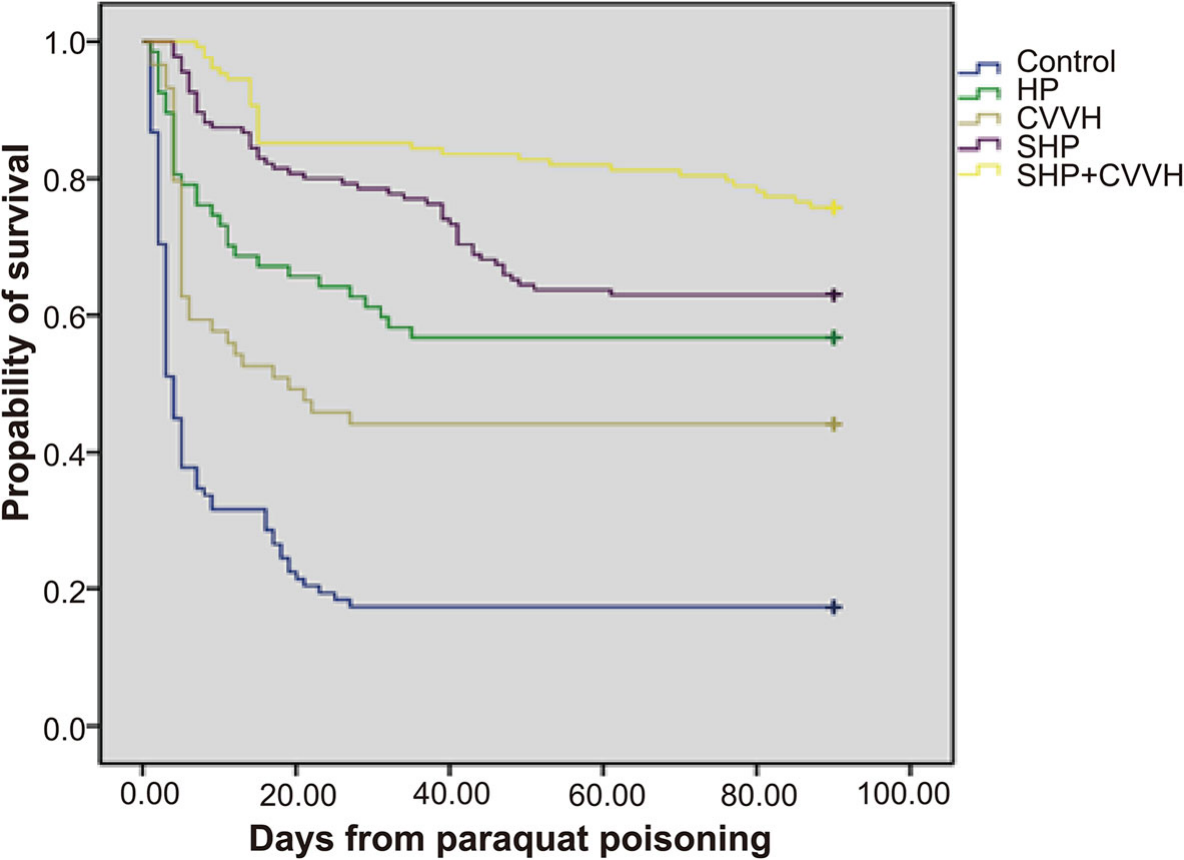
Ninety-day survival curves according to treatment after acute paraquat poisoning. Difference in the survival curves among groups with statistical significance was verified and showed by Log-Rank, χ^2^ = 170.447, *P* = 0.000. Comparison between SHP + CVVH and control: χ^2^ = 118.084, *P* = 0.000; comparison between SHP + CVVH and HP: χ^2^ = 11.003, *P* = 0.001; comparison between SHP + CVVH and CVVH: χ^2^ = 28.549, *P* = 0.000; comparison between SHP + CVVH and SHP: χ^2^ = 5.740, *P* = 0.017

The mortality rates of all groups are depicted in Table 2.On the 90 ^th^ day of follow-up after poisoning, there were 244 deaths among the patients included in the curent research.All cause-mortality rates were 82.27% in control group (81/98), 43.28% in HP group (31/67), 37.04% in SHP group (50/135), 55.93% in CVVH group (33/59), and 24.22% in SHP combined with CVVH group (31/128). The mortality rate of patients in SHP + CVVH was decreased significantly compared with those of other groups (*p* < 0.001). The mortality rate of patients in SHP + CVVH group on the 15 ^th^ day after poisoning was also significantly decreased compared with those of other groups (Table 2). Differences of survival rates among all groups were also statistically significanct (Fig. 2). After calculating the hazards ratio by Cox proportional hazard model, we found that SHP + CVVH was an independent factor reducing the mortality risk (95% CI, 0.107–0.261; *p* < 0.001), (Table 3).

**Table 2:** Mortality rates on the 15th and 90th days after poisoning

	Control (*n* = 98)	HP (*n* = 67)	CVVH (*n* = 59)	SHP (*n* = 135)	SHP + CVVH (*n* = 128)	*P*
Mortality rate, n (%)						
15th day after poisoning	67(68.37)	22 (31.34)a	28 (47.46)	29 (21.48)ac	19 (14.84)abc	<0.001
90th day after poisoning	81 (82.27)	29 (43.28)a	33 (55.93)a	50 (37.04)a	31 (24.22)ac	<0.001

a: compared with the control group, *P*<0.005;

b: compared with the HP group, *P*<0.005;

c: compared with the CVVH group, *P*<0.005

**Table 3:** Risk factors for the death of 90^th^ day in patients with acute Paraquat poisoning assessed by the cox proportional hazard model

Variate	Univariate			Multivariate		
	HR	95%CI	*P*	HR	95%CI	*P*
Gender						
female	Reference					
male	1.016	(0.779, 1.325)	0.905			
Age	1.001	(0.991, 1.011)	0.835			
Poisoning way						
Oral	Reference					
Others	1.198	(0.806, 1.78)	0.372			
Grouping						
Control	Reference			Reference		
HP	0.276	(0.18, 0.423)	< 0.001	0.355	(0.226, 0.557)	< 0.001
CCVH	0.412	(0.274, 0.619)	< 0.001	0.66	(0.438, 0.993)	0.046
SHP	0.205	(0.144, 0.293)	< 0.001	0.359	(0.245, 0.526)	< 0.001
SHP + CVVH	0.124	(0.082, 0.189)	< 0.001	0.167	(0.107, 0.261)	< 0.001
Time from poisoning to treatment	1.004	(0.925, 1.088)	0.933			
Time from poisoning to gastriclavage	1.384	(1.154, 1.659)	< 0.001	1.032	(0.846, 1.257)	0.758
Baseline BUN (mmol/L)	1.063	(1.049, 1.076)	< 0.001	1.066	(1.036, 1.097)	< 0.001
Baseline CREA (μmol/L)	1.001	(1.001, 1.001)	< 0.001	1	(0.999, 1)	0.218
Baseline AST (IU/L)	1.038	(1.033, 1.042)	< 0.001	1.014	(1.007, 1.021)	< 0.001
Baseline ALT (IU/L)	1.045	(1.04, 1.049)	< 0.001	1.019	(1.011, 1.027)	< 0.001
Arterial blood gas analysis						
Baseline PH (mmHg)	0.302	(0.053, 1.731)	0.179			
Baseline PO_2_ (mmHg)	0.894	(0.881, 0.908)	< 0.001	0.943	(0.926, 0.96)	< 0.001
Baseline PCO_2_ (mmHg)	1.007	(0.986, 1.029)	0.499			

### Improvement of organ damage by SHP combined with CVVH

The percentage of patients with organ damage (such as respiratory failure, mechanical ventilation and acute renal injury) in SHP combined with CVVH group was significantly less than that of the other groups (Table 4).

**Table 4:** Organ damage after poisoning

Research results	Control (*n* = 98)	HP (*n* = 67)	CVVH (*n* = 59)	SHP (*n* = 135)	SHP + CVVH (*n* = 128)	*P*
Respiratory failure, n(%)	87 (88.78)	33 (49.25)	42 (71.19)	53 (39.26)a	36 (28.13)ac	<0.001
Mechanical ventilation, n(%)	64 (65.31)	30 (44.78)a	34 (57.63)	53 (39.26)ac	34 (26.56)abc	<0.001
Acute renal injury, n(%)	74(75.51)	44(65.67)	34(50.75)	56(41.48)ab	42(32.81)abc	<0.001

a: compared with the control group, *P*<0.005;

b: compared with the HP group, *P*<0.005;

c: compared with the CVVH group, *P*<0.005

### Influence of SHP combined with CVVH on related inspection indicators

Kidney function of patients with SHP combined with CVVH was significantly superior to that of the other groups. The minimum partial arterial oxygen pressure was higher than that of the other groups and the difference was statistically significant, while the minimum PH value and the maximum partial pressure of carbon dioxide showed no obvious differences in all the groups (Table 5). The related inspection indicators were detected at the time of admission, on days 7 and 14 after paraquat ingestion.

**Table 5:** Related examination indicators after treatment

Indexes	Control (*n* = 98)	HP (*n* = 67)	CVVH (*n* = 59)	SHP (*n* = 135)	SHP + CVVH (*n* = 128)	*P*
Maximum BUN (mmol/L)	49.96 ± 22.8	37.03 ± 18.3a	45.04 ± 23.90	27.13 ± 14.58abc	13.44 ± 12.05abcd	<0.001
Maximum CREA (μmol/L)	749.01 ± 291.02	411.22 ± 174.40a	504.77 ± 183.30a	241.11 ± 108.23abc	174.26 ± 91.74bcd	<0.001
Maximum AST (IU/L)	88.71 ± 76.63	90.04 ± 70.35	92.05 ± 70.88	84.08 ± 75.54a	87.03 ± 65.45abcd	0.018
Maximum ALT (IU/L)	104.43 ± 62.20	101.99 ± 63.47	103.24 ± 66.41	95.07 ± 73.30	91.47 ± 71.12	<0.001
Arterial blood gas analysis						
Minimum PH (mmHg)	7.24 ± 0.11	7.21 ± 0.16	7.26 ± 0.13b	7.29 ± 0.10b	7.28 ± 0.13	<0.001
Minimum PO_2_ (mmHg)	34.17 ± 24.07	52.22 ± 26.79a	41.03 ± 17.79b	63.71 ± 28.84 ac	72.20 ± 13.49abcd	<0.001
Maximum PCO_2_ (mmHg)	46.43 ± 14.05	47.02 ± 13.99	47.10 ± 13.01	45.07 ± 12.14abc	46.82 ± 11.94abc	<0.001

a: compared with the control group, *P*<0.05;

b: compared with the HP group, *P*<0.05;

c: compared with the HP group, *P*<0.05;

d: compared with the CVVH group, *P*<0.05

## Discussion

In this retrospective study, we examined the effect on survival rate and organ damage of SHP combined with CVVH as compared with other groups. The results showed that SHP combined with CVVH reduced the mortality rate on the 90th day after acute paraquat poisoning, also reduced the proportion of patients with organ damage. The survival curve of patients in SHP combined with CVVH group was obviously improved compared with other groups. SHP combined with CVVH was also an independent factor to reduce the mortality risk. These results suggested that SHP combined with CVVH after acute paraquat poisoning could improve the prognosis significantly.

CVVH was provided immediately to the patient to eliminate poison after 10 h SHP. Our study also showed that SHP combined with CVVH reduced the mortality rate of patients with acute paraquat poisoning significantly. The survival rate of patients in SHP combined with CVVH reached 75.78% within 90 days after poisoning, which was superior when compared to other previous studies that adopted single or repeated HP combined with CVVH for treating acute paraquat poisoning [5, 6, 12]. The survival rate of patients in single or repeated HP combined with CVVH was between 46.2 and 68.8%. In the study by Koo et al. [13], the average dose of taking poison of patients was about 44 ml, which was closest to the dose of taking poison in patients of all groups in this study. Their study revealed that HP combined with CVVH could extend the survival time of patients with acute paraquat poisoning, but the mortality rate was not decreased significantly. Recently, Li et al. [4] and Wang et al. [9] found that repeated HP combined with CVVH treatment reduced the mortality rate of patients with acute paraquat poisoning. However, paraquat dose in these two researches was less than that used in our study. Taken together, these findings indicated that SHP combined with CVVH may significantly improve the survival rate of patients with acute paraquat poisoning.

Our study showed that SHP combined with CVVH reduced the incidence rate of acute respiratory failure and acute kidney injury. After acute paraquat poisoning, paraquat is distributed to the lungs, kidneys, heart, brain and other tissues rapidly, thus causing multi-organ dysfunction. The paraquat content in the lungs can be raised promptly, reaching up to 10 times of plasma. Patients with acute paraquat poisoning usually die rapidly due to respiratory failure [14]. The mechanism of lung injury caused by acute paraquat poisoning was not fully clear. Previous studies revealed that it may be related to oxidation-reduction system disorder caused by oxygen-free radicals, HO-1 and other genetic expression changes, cell apoptosis, increased inflammatory factors and other multiple factors [15–18]. Respiratory failure is one of the most common clinical manifestations at the time of acute lung injury. Multiple studies reported that respiratory failure as one of the important factors that influence the prognosis of patients with acute paraquat poisoning [19, 20]. Wu et al. [20] showed that there are some patients with acute paraquat poisoning in Taiwan from 1997 to 2009. Their study included a total of 1811 patients with paraquat poisoning, where 1018 patients had respiratory failure (56.2%) and morality rate of these patients reached up to 93.3%. The findings of this research suggested that patients with acute kidney injury after paraquat poisoning are more likely to have respiratory failure [21]. Our research found that kidney function in SHP combined with CVVH group was superior to that of patients in other groups. Also HP combined with CVVH, extracorporeal membrane oxygenation, immunosuppressor and others might be considered as effective methods for treating respiratory failures caused by acute paraquat poisoning [20, 22]. These findings indicated that SHP combined with CVVH may significantly alleviate organ injury in patients with acute paraquat poisoning.

SHP combined with CVVH may play a role by continuously reducing paraquat content in plasma and eliminating inflammatory factors. Paraquat content in plasma after acute paraquat poisoning reached its peak within short time [3]. Early HP eliminated poisons with middle and small molecular weight. Some scholars hypothesized that HP treatment in the early stage of poisoning can improve the prognosis of patients with paraquat poisoning [3]. However, after poisoning, paraquat accumulates in lungs, muscle, kidneys and other tissues apart from plasma. Paraquat in tissues could be subsequently released into plasma. Early and single HP treatments were difficult to reach the effect of eliminating paraquat effectively. Animal experiments showed that only SHP can significantly improve the survival rate of animals with acute paraquat poisoning [10]. Clinical research found that the use of SHP therapy within 15 h after poisoning improved the survival rate of patients with acute paraquat poisoning [3]. Recently, Chinese scholars also reported that the use of multiple HP treatments could improve the prognosis of patients according to paraquat content in the urine of patients [5]. At present, it was regarded that the inflammatory factor might be one of the important factors for organ damage after acute paraquat poisoning [23]. CVVH not only can eliminate inflammatory factors, but also has slight influence on the hemodynamics. It could eliminate the inflammatory factors continuously, maintain the water electrolyte balance and has other advantages.

However, our study has some limitations. Firstly, this is a retrospective and single-center study, where it involves inherent biases and limitations. Secondly, the therapeutic method is not only influenced by the condition of patients, but also by the intellectual level, economic factors and patients’ families. Thirdly, the serum and urine paraquat levels were not monitored. So, the detection method requires improvement.

## Conclusion

In conclusion, our results showed that SHP combined with CVVH might reduce the mortality rate and be an effective method, alleviating organ damage. However, the research results still need to be verified by multi-center and prospective clinical research studies.

## Data Availability

The data set supporting the results of this article are included within the article.

## Abbreviations

SHP: Strengthened hemoperfusion
CVVH: Continuous venovenous hemofiltration
HP: Hemoperfusion
ANOVA: Analysis of variance
